# Analysis of adrenalectomy for the treatment of adrenal diseases performed by the Public Health Service in São Paulo between 2008 and 2019

**DOI:** 10.1590/0100-6991e-20223320-en

**Published:** 2022-08-01

**Authors:** GUILHERME MIRANDA ANDRADE, ANTONIO OTEROL GIL, ALAN ROGER GOMES BARBOSA, SAULO BORBOREMA TELES, BRENO SANTOS AMARAL, JOSE MONTEIRO, MARCELO APEZZATO, BIANCA BIANCO, GUSTAVO CASERTA LEMOS, ARIE CARNEIRO

**Affiliations:** 1 - Hospital Israelita Albert Einstein, Urologia - São Paulo - SP - Brasil

**Keywords:** Adrenalectomy, Adrenal Gland Diseases, Treatment Outcome, Health Care, Adrenalectomia, Resultado do Tratamento, Assistência Médica, Doenças das Glândulas Suprarrenais

## Abstract

**Introduction:**

treating benign (hormonally active or nonfunctional) and malignant adrenal cancer includes adrenalectomy. The expertise of surgeons and surgery performed by high-volume surgeons were associated with fewer complications and lower cost. We aimed to describe and compare the number of surgeries, mortality rate, and length of hospital stay for adrenalectomies performed between 2008 and 2019 in the public health system of São Paulo.

**Methods:**

this was an ecological study. The data were collected using the TabNet Platform of the Unified Health System Department of Informatics. Outcomes analyzed included the number of surgeries performed, mortality rate during hospital stay, and length of hospital stay. Public hospitals in Sao Paulo were divided into three subgroups according to the surgical volume of adrenalectomies performed as well as hospitals with and without a residency program in Urology, and the results were compared among them.

**Results:**

a total of 943 adrenalectomies were performed in Sao Paulo between 2008 and 2019. Mortality rates during hospital stay according to hospital surgical volume were no reported deaths in low-volume, 0.015% in intermediate-volume, and 0.004% in high-volume hospitals. The average length of the ICU stay was 1.03 days in low-volume, 2.8 in intermediate-volume, and 1.12 in high-volume hospitals (analysis between intermediate and high volume centers with statistical significance, p=0.016).

**Conclusions:**

despite no statistically significant differences among the groups analyzed, mortality rates were very low in all groups. ICU stay was shorter in high-volume centers than in intermediate-volume centers.

## RESUMO

**Introdução:**

o tratamento do câncer de adrenal benigno (hormonalmente ativo ou não funcional) e maligno inclui a adrenalectomia. A experiência dos cirurgiões e a cirurgia realizada por cirurgiões de alto volume foram associadas a menos complicações e menor custo. O objetivo do estudo foi descrever e comparar o número de cirurgias, a taxa de mortalidade e o tempo de internação para adrenalectomias realizadas entre 2008 e 2019 na rede pública de saúde de São Paulo.

**Métodos:**

trata-se de um estudo ecológico. Os dados foram coletados da Plataforma TabNet do Departamento de Informática do Sistema Único de Saúde. Os hospitais foram divididos em três subgrupos de acordo com o volume cirúrgico e hospitais com e sem programa de residência médica em Urologia. Os resultados foram comparados entre os grupos.

**Resultados:**

no período estudado, 943 adrenalectomias foram realizadas em São Paulo. As taxas de mortalidade durante a internação de acordo com o volume cirúrgico hospitalar foram: não foram relatados óbitos em hospitais de baixo volume; 0,015% em hospitais de volume intermediário e 0,004% em alto volume. O tempo médio de permanência na UTI foi de 1,03 dias nos hospitais de baixo volume; 2,8 nos de médio volume e 1,12 nos de alto volume (análise entre centros de médio e alto volume com significância estatística, p=0,016).

**Conclusão:**

as taxas de mortalidade foram muito baixas em todos os grupos. A permanência na UTI foi menor em centros de alto volume do que em centros de volume intermediário.

## INTRODUCTION

Adrenal tumors are usually asymptomatic and, thus, are frequently diagnosed through abdominal imaging done for unrelated reasons. Incidentalomas are found in up to 9% of autopsy cases and in 4% of imaging series^1^. Approximately 54% of adrenal incidentalomas are adrenal adenomas, which are more prevalent in women than men (55% vs 45%)^2^, and the prevalence increases with age, with a probability of 0.2% in the third decade to 7% in the eighth decade^1^. Another reference shows an adrenal cancer incidence of 2 per million, and it is responsible for 0.2% of all cancer deaths^3^. 

Hormonal assessment is mandatory after diagnosis to prove or exclude overproduction of cortisol, aldosterone, or catecholamines to differentiate functioning from non-functioning masses. Literature shows that complication rates - glucose intolerance, cardiovascular disease, and dyslipidemia - are comparable between patients with functional and non-functional adrenal tumors^4^. 

Hormonal production by an adrenal mass has a genetic cause, but it is not entirely clear^2^. Most adrenal tumors are benign and non-functioning adenomas. Others benign adenoma tumors are secretors of hormones (15%) that course, for example, with Cushing’s syndrome (1-29% average 9%), primary aldosteronism (1.5-3.3%) and pheochromocytoma (1.5-11%)^2^. 

Few adrenal tumors are malignant. The majority of pheochromocytomas (PCC) are benign, but up to 25% may be malignant, which is a rare neuroendocrine malignancy arising from neural crest adrenal medulla cells. It is estimated with an annual incidence of 0.8 per 100,000 people, and approximately 500-1,600 new cases per year in the United States. PCC is highly vascular and secretes catecholamine, presenting with the over-activation of the sympathetic nervous system^5^. Diagnosis is confirmed by raised plasma or urine metanephrines or normetanephrines. Radiology assists in the tumor location and any local invasion or metastasis. All patients should undergo preoperative preparation with α-blockers and/or other medications to control hypertension, arrhythmia, and volume expansion^6^. 

Adrenocortical carcinomas have a lower incidence, with a more aggressive presentation and high mortality rate^7^, which can be functional or non-functional^8^. Rarely, both adrenal glands may be affected. In these cases, the main causes are metastatic disease, congenital adrenal hyperplasia, lymphoma, infections, or hemorrhage^2^. 

Treating benign (hormonally active or nonfunctional) and malignant adrenal cancer includes adrenalectomy. The presence of symptoms or biochemically functioning tumors is an indication for surgical intervention in adrenal masses. If asymptomatic, the size (<4cm) and imaging characteristics (<10UH) may be indications for observation management^1^ ^,^ ^9^. 

Adrenal carcinoma is more likely in larger lesions, with tumors larger than 6cm having a 19% chance of malignancy, reaching 47% in lesions larger than 8cm^10^. In fact, in terms of cost effectiveness, adrenal tumors larger than 4cm, even though they are not functioning, should be treated surgically because of the possibility of the presence of carcinoma and the aggressiveness of the disease with adjuvant treatments with low effectiveness^11^. 

The prevalence of secondary arterial hypertension varies from 3% to 5%. Parenchymal kidney disease and sleep apnea are the most common causes of secondary hypertension. Other causes include renovascular, primary hyperaldosteronism, pheochromocytoma, aortic coarctation, Cushing’s syndrome, hypothyroidism, hyperthyroidism, hyperparathyroidism, and acromegaly^12^.

Primary hyperaldosteronism is a disease characterized by inappropriate aldosterone secretion and plasma renin suppression. The most common causes of primary hyperaldosteronism are aldosterone-producing adenomas and bilateral adrenal hyperplasia, but they can also be caused by adrenal carcinoma and extra-adrenal tumors that produce aldosterone. Conventionally, the prevalence of primary hyperaldosteronism is not highly uncommon, and hyperaldosteronism is present in about 20% of the population of resistant hypertensive patients^13^. In a follow-up of 600 hypertensive patients, the prevalence of primary hyperaldosteronism was 6.1%^14^.

It is interesting to note that the meta-analysis by Bancos et al. demonstrated benefits after adrenalectomy in the control of cardiovascular risk factors in patients with adrenal tumors when compared to conservative treatment^15^. In contrast, the effect of adrenalectomy on the control of cardiovascular risks in patients with Cushing’s syndrome still needs further investigation and studies^16^. 

The access used to perform an adrenalectomy can be: transabdominally, retroperitoneally, or transthoracic. The technique commonly used is transabdominal adrenalectomy by open or minimally invasive surgery (MIS)^17^. Laparoscopic adrenalectomy has become the gold standard since it was reported by Gagner in 1992^18^. The advantages of this technique include decreased postoperative pain, fewer complications, shorter hospital stay, faster recovery, cost-effectiveness, and cosmetics^1^ ^,^ ^19^ ^,^ ^20^.

To minimize the intraoperative complications of an adrenalectomy, we must perform extensive exposure and visualization of the operative field and isolating the main vascular structures and resection using the block technique to prevent capsular rupture^21^. Choosing a particular surgical technique involves the surgeon’s experience, size of the lesion, and involvement of adjacent structures^22^. The mortality rate of adrenalectomy is very low, and morbidity rates vary from 6% to 30%^23^.

The expertise of surgeons and surgery performed by high-volume surgeons were associated with fewer complications and lower cost^24^. In Brazil, the majority of patients rely on public health services, also known as SUS (Sistema Único de Saúde) for medical treatment. São Paulo has the 8^th^ largest population worldwide, and it is estimated that in 2016, it had approximately 12 million inhabitants and almost 5 million of them relied on SUS.

On the other hand, the role of radiation and chemotherapy is limited, and the effect of adjuvant mitotane is unproven^25^. For patients with localized disease at presentation, oncologic outcome and the success of surgical therapy are dependent on the completeness of resection of the primary tumor, the surrounding retroperitoneal tissue, and the regional lymph nodes^26^.

The objective of this study was to describe and compare the number of surgeries, mortality rate during hospital stay, length of hospital stay, ICU stay, and costs of adrenalectomies performed by SUS between 2008 and 2019 in São Paulo city according to hospital surgical volume and the difference between hospitals with and without residency programs.

## METHODS

This is an ecological study that analyzed data available from the TabNet platform of the Unified Health System Department of Informatics (DATASUS), which provides open data on procedures performed through the Brazilian public health care system. Data are unidentified on the platform; informed consent was not feasible and therefore not requested by the local ethics committee. The study was approved by the Ethics Committee at Hospital Israelita Albert Einstein (number CAAE: 17208019.0.0000.0071).

We examined three procedural codes of the Unified Health System Table of Procedures, Medications, Orthoses, Prostheses, and Materials Management System (SIGTAP/SUS) for the treatment of adrenal diseases: “bilateral adrenalectomy” - code 0402020014, “unilateral adrenalectomy” - code 0402020022 and “adrenalectomy in oncology - code 0416010202” 

All information was retrieved from publicly available websites using web scraping software. Coding was performed in Python v. 2.7.13 (Python Software Foundation, Beaverton, OR, USA) running on a Windows 10 Single Language operating system (Microsoft Corporation, Redmond, WA, USA). Data collection, field selection, and table sorting were performed using open-source packages selenium-webdriver v. 3.1.8 (Selenium HQ, various developers worldwide) and pandas v. 2.7.13 (Lambda Foundry, Inc. and PyData Development Team, New York, NY, USA). 

The web scraping code had a main structure with 14 adaptive search steps (Appendix A) for the different filters available on the platform. We used Mozilla Firefox v. 59.0.2 browser (Mozilla Corporation, Mountain View, CA, USA) and GeckoDriver v. 0.18.0 webdriver (Mozilla Corporation, Bournemouth, England). 

Following data collection, standard procedures of data transformation and cleaning were performed for each file, including the removal of header and footer information, removal of the health facility code, and conversion of date columns to rows. The data were saved and stored in Microsoft Office Excel 2016^®^ v. 16.0.4456.1003 spreadsheet (Microsoft Corporation). The following information was extracted from the TabNet dataset: total number of adrenalectomies performed, mortality rate during hospital stay, and length of hospital stay and costs. 

The public hospitals of São Paulo were divided into three subgroups according to the volume of surgeries performed (tercil), and the results were compared among themselves. Nine centers were included as low volume, performing 1 to 3 surgeries. Another nine hospitals classified in the intermediate group performed between 4 and 14 surgeries. Finally, eight centers were included as high volume and performed between 29 and 470 surgeries. Hospitals were also divided into two groups: with and without a residency program in Urology and the results were compared as well.

Statistical analysis was performed using SPSS v. 24 (SPSS for Mac OS X, SPSS, Inc., Chicago, IL, USA). Quantitative variables were analyzed with the Mann-Whitney U test or T test according to the Kolmogorov-Smirnov test for normality. Proportions were analyzed using the chi-square test. Statistical significance was determined at p<0.05.

## RESULTS

**Figure 1:**
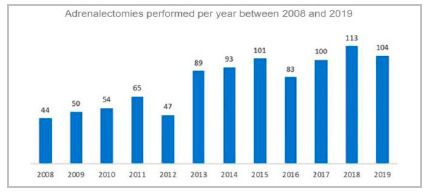
Adrenalectomies performed per year between 2008 and 2019 in São Paulo.

A total of 943 adrenalectomies were performed in São Paulo city between 2008 and 2019 in 26 hospitals in São Paulo. The number of surgeries performed increased over the years, especially after 2012. The lowest number of surgeries was in 2008, with 44 procedures and the highest in 2018, with 113 (Figure 1).

Global mortality during hospital stay was very low, with 5 deaths among 943 surgeries (0.005%). The mortality rate divided by hospital volumetry is shown in Table 1. None of the analyses showed statistically significant differences between the groups. High-volume (HV) hospitals had a mortality rate of 0.004%, while intermediate volume (IV) centers had a 0.015% mortality rate. There were no reported deaths in low-volume hospitals (Table 1). 

**Table 1:** Length of hospital stay, ICU hospitalization, hospital mortality, and costs among low, intermediate, and high-volume centers.

Variables	Low volume	Intermediate volume	High volume	p-value
Length of hospital stay, days (Mean [SD])	5.47 (3.39)	13 (12.16)	6.35 (1.83)	0.041^a^
				0.083^b^
				0.335^c^
ICU hospitalization, days (Mean [SD])	1.03 (1.41)	2.80 (2.51)	1.12 (0.56)	0.089^a^
				0.464^b^
				0.016^c^
In-hospital mortality (n [%])	0/14 0%	1/65 0.015%	4/864 0.004%	0.638^a^
				0.794^b^
				0.254^c^
Costs, dólar (Mean [SD])	237 (125)	536 (237)	438 (122)	0.004^a^
				0.005^b^
				0.309^c^

^a^p-value of the comparison between low and intermediate volume centers; ^b^p-value of the comparison between intermediate and high-volume centers; ^c^p-value of the comparison between low and high-volume centers.

The average length of hospital stay per surgery was 5.47 days in low volume, 13 days in intermediate volume, and 6.35 days in high-volume hospitals, with statistical significance when comparing low with intermediate volume centers (p=0.041) (Table 1). Considering the length of ICU stay, the average number of days per surgery was 1.03 in low-volume centers, 2.8 in intermediate volumes, and 1.12 in high-volume centers. Analysis between the groups showed a significant difference between intermediate and high-volume centers (p=0.016) (Table 1).

The lowest volume center was related to less cost compared to higher volume centers ($237 vs. $536, p=0.004 for low vs. intermediate volume and $237 vs. $438, p=0.005, for low vs high volume) (Table 1).

Comparing institutions with residency programs in Urology and without residency programs in Urology, length of hospital stay, ICU hospitalization, mortality, and costs did not show significant differences between both groups (Table 2).

**Table 2:** Length of hospital stay, ICU hospitalization, in-hospital mortality, and costs between institutions with and without residency program in São Paulo between 2008 and 2019.

Variables	Academic Centers	Non-Academic Centers	p-value
Length of Hospital Stay, days (mean [SD])	7.50 (6.63)	9.33 (9.56)	0.661
ICU hospitalization, days (mean [SD])	1.92 (2.32)	1.38 (1.12)	0.959
In-hospital mortality (n [%])	4/851 (0.4%)	1/92 (1%)	0.441
Costs, Reais R$ (mean [SD])	2,303.71 (1,335.05)	2,108.80 (941.07)	0.676

## DISCUSSION

The adrenal gland produces hormones fundamental to the functioning of the organism with the production of aldosterone, cortisol, and androgenic hormones being in the cortical, noradrenaline, and adrenaline being in the marrow. The uncontrolled production of these hormones triggers clinical complications with a high rate of morbidity and mortality when not properly treated. 

Our public health system has shown an increase in the number of patients undergoing adrenalectomy in recent years, with greater access by the population to modern diagnostic tests. However, there is still an underreporting of patients who could benefit from the treatment of adrenal tumors.

Another aspect shown in our series is the prolonged period of hospitalization of operated patients, probably related to the lack of knowledge of the true nature of pathologies related to the adrenal gland and the lack of coordination between clinical, surgical, and anesthesia teams, due to the metabolic changes that these patients present, requiring multidisciplinary treatment.

This is the first Brazilian study based on information extracted from a public health system database of a low-to-middle-income country that compared the outcomes of adrenalectomies according to hospital surgical volume. All data analyzed in our study are publicly available and were collected from the Unified Health System Department of Informatics (DATASUS), created in 1991 to provide SUS with information systems and computer support services. 

The Unified Health System Table of Procedures, Medications, Orthoses, Prostheses, and Materials Management System (SIGTAP/SUS) provides information on which SUS-affiliated institutes are qualified to perform each procedure and help improve financial decision-making.

When we analyzed adrenal diseases in our city, we did not identify differences between hospitals with and without a residency program when we compared the following aspects: length of hospital stay, ICU hospitalization, intrahospital mortality, and costs. Perhaps this can be explained by the study by Park et al., who identified that the main factor related to complications is the volume of surgeries performed by the surgeon. In hospitals with medical residency, we have residents with little surgical experience performing adrenalectomies guided by experienced surgeons while in hospitals without medical residency, surgeons with high surgical baggage (ability, experience, and technical skills) may also be present, which can match the results^27^.

The authors indicated the operative time and intraoperative complications reduction accruing the experience, leading to locating the flattening of the learning curve after approximately 30 cases were performed. Eto et al. reported a shorter learning curve for surgeons after participating in at least 10 procedures as an assistant surgeon^21^ ^,^ ^28^.

Another explanation for the lack of difference between teaching and non-teaching hospitals is that usually the most complex cases or those involving malignant neoplasms are referred to university centers, while simpler cases are often performed in smaller hospitals. This study presents a bias for not stratifying the reason for the adrenalectomy performed by each service.

It was observed that adrenal surgeries performed by higher volume centers are associated with fewer unfavorable clinical outcomes compared to intermediary volume centers. These observations are in agreement with previously published findings by other investigators. According to Al-Qurayshi Z et al., the centralization and selective referral of patients who require adrenalectomy to high-volume surgeons holds conceivable savings potential for patients overall^24^. Another advantage of operating on these patients in referral centers is perioperative anesthetic care that requires knowledge and expertise in dealing with possible pressure changes and hemodynamic instability secondary to the systemic release of hormones^28^.

Several studies support the importance of referral centers in accomplishing the learning curve. Although in recent years, thanks to the improvement of diagnostics, the number of adrenal pathologies requiring surgery has greatly increased, they still have a low incidence, and adrenal surgery is still rarely performed in non-referral centers^28^.

Other studies have worked with thousands of people^29^. The limited number of participants may represent a bias in this study. The low number of surgeries in the low surgical volume group may mask the detection of operative complications, which explains the lack of statistical difference between the results of centers with low and medium surgical volume. Costs were lower in the low-volume center probably because simpler cases are often performed in smaller hospitals without the need for postoperative ICU stay, while the most complex cases or those involving malignant neoplasms are referred to university centers with higher surgical volume.

Nonetheless, Kazaure and Sosa^30^ observed that when compared to low-volume surgeons, high-volume surgeons on average achieve lower rates of postoperative complications and mortality, as well as a shorter length of hospital stay, and lower cost of hospitalization. However, no similar association between hospital adrenalectomy volume and improved patient outcomes were found. Park HS et al. observed that surgeon volume, specialty, and hospital volume were not predictors of costs^27^.

Currently, 9% of hypertension cases are attributed to secondary endocrine causes. In fact, hypertension is curable surgically in a minority of patients, especially in patients with aldosterone-secreting adenomas or with functional pheochromocytomas or paragangliomas^31^. The low volume of adrenalectomies performed in hospitals in São Paulo corroborates the hypothesis that many cases of secondary hypertension with surgical indications are not adequately treated.

This study has some limitations, such as data source, which was restricted to the SUS database, depending on the notification of the procedures and outcomes by each institution. In the database system, we had access to limited outcomes. The DATASUS depends on the proper filling of data by health professionals, in addition this database does not cover primary care, only secondary and tertiary care, so some important information about patients’ demographics, comorbidities, and symptoms or tumor type and location, and anatomopathological exams are not provided. 

## CONCLUSION

The number of adrenal surgeries has increased over the years. Despite no statistically significant differences among the groups analyzed, mortality rates were very low in all groups. ICU stay was shorter in high-volume centers than in intermediate-volume centers. Costs were lower in low-volume centers.
